# Comparing EQ-5D-5L, PROPr, SF-6D and TTO utilities in patients with chronic skin diseases

**DOI:** 10.1007/s10198-024-01728-5

**Published:** 2024-09-28

**Authors:** Ákos Szabó, Valentin Brodszky, Fanni Rencz

**Affiliations:** 1 Department of Health Policy, Corvinus University of Budapest, 8 Fővám tér, Budapest, H-1093 Hungary; 2 Károly Rácz Doctoral School of Conservative Medicine, Semmelweis University, 26 Üllői út, Budapest, H-1085 Hungary

**Keywords:** EQ-5D-5L, SF-6D, PROPr, TTO, Utility, Health-related quality of life, Validity, Psychometrics, I10

## Abstract

**Objectives:**

We aim to compare the measurement properties of three indirect (EQ-5D-5L, PROPr, SF-6D) and one direct (time trade-off, TTO) utility assessment methods in patients with chronic skin diseases.

**Methods:**

120 patients with physician-diagnosed chronic skin diseases (psoriasis 39%, atopic dermatitis 27%, acne 19%) completed a cross-sectional survey. Respondents completed the EQ-5D-5L, PROMIS-29+2 and SF-36v1 questionnaires and a 10-year TTO task for own current health. Utilities were computed using the US value sets. Ceiling, convergent and known-group validity were compared across the utilities derived with these four methods. Known-groups were defined based on general, physical and mental health. The agreement between utilities was assessed using intraclass correlation coefficients (ICC).

**Results:**

Mean utilities for the EQ-5D-5L, PROPr, SF-6D and TTO were 0.79, 0.47, 0.76 and 0.89. In corresponding order, the ceiling was 28%, 0%, 2% and 65%. The SF-6D showed excellent agreement with the EQ-5D-5L (ICC = 0.770). PROPr demonstrated poor agreement with the EQ-5D-5L (ICC = 0.381) and fair with SF-6D utilities (ICC = 0.445). TTO utilities showed poor agreement with indirectly assessed utilities (ICC = 0.058–0.242). The EQ-5D-5L better discriminated between known groups of general and physical health, while the SF-6D and PROPr outperformed the EQ-5D-5L for mental health problems.

**Conclusion:**

There is a great variability in utilities across the four methods in patients with chronic skin conditions. The EQ-5D-5L, despite its higher ceiling, appears to be the most efficient in discriminating between patient groups for physical health aspects. Our findings inform the choice of instrument for quality-adjusted life year calculations in cost-utility analyses.

**Supplementary Information:**

The online version contains supplementary material available at 10.1007/s10198-024-01728-5.

## Introduction

Chronic skin diseases represent a substantial burden on patients, society, and health systems [1–3]. Skin conditions accounted for 1.79% of the overall global disease burden, as quantified in Disability-Adjusted Life Years across 306 diseases and injuries, making them the 4th leading cause of non-fatal disease burden [4, 5]. Chronic skin diseases, such as psoriasis, atopic dermatitis, urticaria or vitiligo often negatively affect patients’ health-related quality of life (HRQoL); yet they are rarely life threatening. Consequently, health policy decision-makers may perceive them as less severe than other health problems. However, an increasing number of novel, high-cost treatments, such as biological agents and small molecule therapies, are becoming available for the treatment of chronic skin conditions, which require economic evaluations, such as cost-utility analyses [6].

Cost-utility analysis aims to determine the cost of a quality-adjusted life year (QALY) [7]. QALY is a single metric of health gain that combines life expectancy (survival) and HRQoL (utility) improvements. Utilities represent preferences for health states, where full health is assigned a value of 1, while a health state as bad as being dead is assigned a value of 0. Negative utilities refer to health states considered as worse than being dead [8, 9]. Utilities may be measured directly or indirectly using various approaches. Direct assessment methods include the time trade-off (TTO) and standard gamble, among others, where respondents express their preferences by making decisions between two alternatives, such as living a certain number of years in an impaired health state versus living a shorter life but in full health. The most widely used direct utility assessment method is the TTO, which may be used to value hypothetical health states as well as own health [10].

Indirect measures involve using generic or disease-specific preference-accompanied HRQoL questionnaires. These measures typically comprise two components: a descriptive system and a value set, which allows assigning utilities to each health state described by the descriptive system [11, 12]. Over the last decades, the number of generic preference-based measures, such as the EQ-5D, Short-form 6-dimensions (SF-6D), 15D, Assessment of Quality of Life, or Patient-Reported Outcomes Measurement Information System-Preference (PROPr), has increased [13].

There has been an increasing need for health utility assessments in chronic skin conditions due to the availability of new treatments [14–16]. However, there is no specific measurement approach recommended for this area. Generally, the EQ-5D and direct TTO utilities are the most commonly used in chronic skin conditions [17, 18]. A recent study developed a TTO-based experimental value set for the most commonly used skin-specific measure, the Dermatology Life Quality Index (DLQI) [19]. Furthermore, there are existing mapping algorithms in the literature that convert DLQI responses or scores to EQ-5D utilities [20–23]. Few studies have so far used other generic preference-accompanied measures than the EQ-5D, such as the SF-6D and the 15D in this context [24–26].

Different measurement methods can yield varying utilities and improvements achievable with treatments. These variations directly impact incremental cost-utility ratios and, consequently, resource allocation decisions in healthcare. Instruments assess different dimensions and use various preference elicitation methods, which affect these outcomes and the sensitivity of the methods to the symptoms experienced by specific patient populations. Not all clinical trials use all available instruments; therefore, understanding the differences between them is crucial, for example, when assessing their interchangeability. Certain instruments may be insensitive in some populations, leading to recommendations against their use. Multi-instrument comparison studies can provide valuable evidence to help select and support the use of certain measurement methods. Such studies have previously been conducted in samples involving the general population and in specific clinical areas, such as rare diseases, multiple sclerosis and chronic kidney disease [27–31]. Although most of the abovementioned measurement methods have been used to estimate utilities in various chronic skin disease populations, there is very limited comparative evidence available about their psychometric performance in chronic skin conditions. Previous comparative studies have primarily focused on the comparison between the three-level (EQ-5D-3L) and five-level EQ-5D (EQ-5D-5L), with limited information on comparisons with other utility assessment methods in this specific context [32–34].

Our aim therefore was to compare the measurement properties of three indirect (EQ-5D-5L, PROPr and SF-6D) and one direct (TTO) utility assessment methods in patients with chronic skin diseases.

## Methods

### Study design and population

An online cross-sectional study was conducted among a representative sample of the general adult population in Hungary in November 2020, after receiving approval from the Research Ethics Committee of the Corvinus University of Budapest (reference No. KRH/343/2020). A detailed description of the data collection is available elsewhere [27, 35–38]. A survey company recruited all participants from the members of the largest Hungarian online panel. We used three inclusion criteria: (1) being aged 18 years or older, (2) literacy in Hungarian, and (3) providing informed consent to participate in the study. All respondents could complete the questionnaire only once, and received survey points as a compensation for participation. The present study uses data from 120 respondents out of the total sample of 1,700, all of whom reported having a chronic skin condition.

### Survey instrument

Participants were asked to complete a self-administered questionnaire which was intended to measure HRQoL and wellbeing. The questionnaire included the official Hungarian-language versions of the EQ-5D-5L, PROMIS-29+2 v2.1, Short Form 36 Health Survey (SF-36v1), PROMIS Global Health v1.2 and a TTO task, presented in a fixed order. The survey instrument also collected the respondents’ sociodemographic information (age, gender, employment status, highest level of education and place of residency) and existing health conditions. Information regarding chronic health conditions was asked in a two-step process. Initially, respondents were asked to indicate whether they had experienced one or more chronic health conditions or chronic consequences of acute conditions in the past 12 months, using a predefined list of prevalent chronic diseases [39]. In the subsequent step, respondents were asked to specify conditions that had been diagnosed by a physician. The predefined list included the most prevalent non-dermatological diseases (e.g. cardiovascular disease, musculoskeletal disease, diabetes), along with the following skin diseases: psoriasis, atopic dermatitis, acne, hidradenitis suppurativa and vitiligo. Additionally, respondents had the opportunity to report any additional (skin) conditions under an ‘other’ category. Since all questions were mandatory, there were no missing data.

### Outcome measures

We used one direct (TTO) and three indirect methods (EQ-5D-5L, PROPr and SF-6D) to measure the health utilities (Table 1). For the sake of consistency, we used the US value set for each questionnaire. However, we have repeated some of the analyses using the Hungarian EQ-5D-5L value set to allow for more direct comparisons with the TTO utilities derived from Hungarian respondents. Additionally, the questionnaire included the PROMIS Global Health questionnaire that was used for the known group validity tests.

**Table 1:** Characteristics of health utility measures used in the study

	EQ-5D-5L		SF-6Dv1	PROPr	TTO	
Questionnaire	EQ-5D-5L		SF-36v1	PROMIS-29+2 v2.1	n/a	
Type of questionnaire	generic		generic	generic	n/a	
Health domains included	1) mobility 2) self-care 3) usual activities 4) pain / discomfort 5) anxiety / depression		1) physical functioning 2) role limitations 3) social functioning 4) pain 5) mental health 6) vitality	1) physical function 2) depression 3) fatigue 4) sleep disturbance 5) ability to participate in social roles and activities 6) pain interference 7) cognitive function	n/a	
Response levels	5-level severity scale		4/5/6-level severity/frequency/interference with functioning scale	5-level severity/frequency/interference with functioning/global rating/capability scale	21 possible answers ranging from 0 yrs to 10 yrs with 6-month steps	
Recall period	today	4 weeks / unspecified		7 days / unspecified	unspecified	
Number of health states	3,125	18,000		217,238,121	n/a	
Value set	United States	United States		United States	n/a	
Valuation method	composite TTO (EQ-VT 2.1)	Paired comparisons (standard gamble)		standard gamble	Conventional TTO	
Perspective of utilities	societal	societal		societal	individual	
Range (min to max) *	-0.573 to 1.0	0.013 to 1.0		-0.022 to 0.954	0 to 1.0	

SF-6D = Short-form 6-dimensions; SF-36 = Short-Form-36; PROPr = Patient-Reported Outcomes Measurement Information System-Preference; TTO = Time trade-off

* The maximum value of the range represents full health, while 0 refers to health states as bad as being dead. Negative values in EQ-5D-5L and PROPr represent health states valued to be worse than being dead

#### Time trade-off (TTO)

Respondents were asked to rate their current own health using a 10-year conventional TTO question. We chose to use a 10-year time frame, as this duration is widely used in both vignette-based and value set development studies in Hungary and elsewhere [19, 40–44]. Participants were asked the following TTO question: “Imagine that you have exactly 10 more years to live in your current state of health. Or you can choose to live in full health for a shorter period. What is the maximum amount of your remaining 10 years you would be willing to sacrifice to live in full health instead of your current state of health?” The specific response options provided were: none, 6 months, 1 year, 1 year 6 months, 2 years, 2 years 6 months and up to 10 years (equal to immediate death). We did not provide participants with the option to rate their own health as worse than being dead, as it was considered unlikely to be chosen by the majority of respondents in a general population sample. TTO utilities were calculated using the following formula:

$$\:U=\frac{10\:years-responden{t}^{{\prime\:}}s\:answer}{10\:years}$$

For instance, if a patient chose to give up 4 years, the utility (U) would be calculated as (10 − 4) / 10 = 0.6.

#### EQ-5D-5L

The EQ-5D-5L is the most commonly used generic preference-accompanied measure [45]. The instrument assesses the respondents’ health on the day of completion across five different health domains (mobility, self-care, usual activities, pain/discomfort and anxiety/depression). Each domain has five response levels. EQ-5D-5L utilities were estimated using the US value set [46], and in the sensitivity analysis using the Hungarian value set [42]. The questionnaire also contains the EuroQol visual analogue scale (EQ VAS), which assesses on the respondent’s current health status on vertical scale. On this scale, 0 represents ‘the worst health you can imagine’, while 100 indicates ‘the best health you can imagine’.

#### PROPr

PROPr utilities were estimated based on the PROMIS-29+2 v2.1 generic preference-accompanied measure using the US value set [47, 48]. The PROMIS-29+2 questionnaire assesses health across eight domains, including physical function, anxiety, depression, fatigue, sleep disturbance, ability to participate in social roles and activities, pain interference and cognitive function. Additionally, the measure includes a 0–10 numeric rating scale to assess pain intensity. All domains except anxiety are used for the estimation of PROPr utilities. Each domain consists of four items, except for cognitive function, which has two items. Each item is measured on a 5-point scale, except for the abovementioned pain intensity scale.

#### SF-6D

SF-6D utilities were estimated based on the responses on the 36-Item Short Form Survey version 1 (SF-36 v1 [49]) using the US value set [50]. The SF-6D uses responses to a subset of items of the SF-36 questionnaire. The SF-6D includes six health domains (physical functioning, role limitations, social functioning, pain, mental health and vitality), each represented by one item, with four to six response options. Additionally, the first question of SF-36 (“*How would you describe your health?”*) was used for known-groups validity tests.

#### PROMIS Global Health

PROMIS Global Health is a generic health status measure, providing a broad assessment of mental and physical health [51]. It consists of nine items, each rated on a 5-point scale and a pain intensity scale that is identical to the one used in PROMIS-29+2. In this study, we used the first four general global items to define known groups *(Global01 – “In general*,* would you say your health is:”; Global02 – “In general*,* would you say your quality of life”; Global03 – “In general*,* how would you rate your physical health?”; Global04 – “In general*,* how would you rate your mental health*,* including your mood and your ability to think?”)*.

### Statistical analyses

Demographic characteristics were reported as proportions for categorical variables and medians and interquartile ranges for continuous variables. We present the descriptive characteristics of utilities for the total sample and across four skin disease subgroups of patients, including minimum, maximum, mean, standard deviation, median and interquartile range. Histograms were used to visualize the distribution of utilities. The following measurement properties were assessed: ceiling and floor, convergent validity, agreement and known-group validity.

### Ceiling and floor

To investigate ceiling and floor of the utilities, we have analysed the relative frequency of the maximum (ceiling) and minimum (floor) values for each utility assessment method. Ceiling and floor effects were considered to be present if at least 15% of the respondents achieved the maximum or minimum utility value [52]. We hypothesized that EQ-5D-5L and TTO would demonstrate ceiling effect [10, 53]. Considering the target population, we assumed no floor effect on any of the measures.

### Convergent validity

Convergent validity between EQ-5D-5L, SF-6D, PROPr and TTO utilities were evaluated by Spearman’s rank-order correlations. Correlation coefficients (r_s_) below 0.20 were considered very weak; between 0.20 and 0.39 weak, between 0.40 and 0.59 moderate, between 0.60 and 0.79 strong and those ≥ 0.80 very strong [54]. We hypothesized strong correlations between the three indirect utility assessment methods, but weak correlations between the indirect methods and the TTO [27, 55–60].

### Agreement

Intraclass correlation coefficients (ICC) and Bland-Altman plots were used to explore the agreement between the health utilities. ICC was calculated with a two-way random model with absolute agreement, where the interpretation of ICC coefficients was the following: <0.39 (poor); 0.40–0.59 (fair); 0.60–0.74 (good) and 0.75< (excellent) [61, 62]. The Bland-Altman plots were used to display the mean utilities (x-axis) and differences between utilities (y-axis) [63, 64]. Poor or fair agreement was hypothesized between the four utility assessment methods [27, 60].

### Validity between known groups

The non-parametric Kruskal–Wallis H-test was employed to assess known-groups’ validity of the utility assessment methods across subgroups of patients. Known-groups were defined based on the first item of SF-36 and the first four items of PROMIS Global Health. The ‘excellent’ and ‘very good’ categories of each question were combined due to the small number of respondents marking the former. We expected that TTO would result in the highest average utilities, while PROPr would yield the lowest across all subgroups [27, 65]. We hypothesized that the EQ-5D-5L could better discriminate between known groups of patients defined based on general health, physical health, but not for quality of life or mental health. The effect sizes (ES) were estimated for each examined group according to the following formula:

$$\:ES\left(H\right)=\:\frac{Kruskal-Wallis\:H-k+1}{n-k}$$,

where *k* indicates the number of groups and *n* denotes the size of the sample. ES values were interpreted as small if were ≥ 0.01, moderate if ≥ 0.06 and large if ≥ 0.14 [66, 67]. Furthermore, relative efficiencies were computed as the ratio of the ESs of two selected utility assessment methods. For relative efficiency computations, the EQ-5D-5L was selected as the reference measure (i.e. denominator). A relative efficiency value of > 1.0 would indicate that a utility assessment method is more efficient in differentiating between known groups than the EQ-5D-5L.

All the statistical tests were two-sided, and *p* < 0.05 was considered statistically significant. All analyses were conducted using IBM SPSS Statistics for Windows software, Version 27.0 (IBM Corp, Armonk, New York, NJ, USA), while all plots were created with R Statistical Software (version 4.1.1; R Foundation for Statistical Computing, Vienna, Austria).

## Results

### Characteristics of the study population

A total of 120 patients were included in our study (Online Resource 1). The median age was 51 years (minimum 18, maximum 86 years). More than half of the sample were female (*n* = 73, 61%). The majority had secondary or higher education level (*n* = 84, 70%), and 41% were employed full-time or part-time. The following self-reported, physician-diagnosed chronic dermatological conditions occurred in the sample: psoriasis (*n* = 47, 39%), atopic dermatitis (*n* = 32, 27%), acne (*n* = 23, 19%) and other (*n* = 24, 20%). The median (IQR) EQ VAS score was 80.00 (69.25-89.00). Based on the first question of the SF-36, 23% of the sample reported being in excellent or very good health, 42% in good health, 25% in poor health, and 11% in very poor health.

### Descriptive results, ceiling and floor

**Fig. 1:**
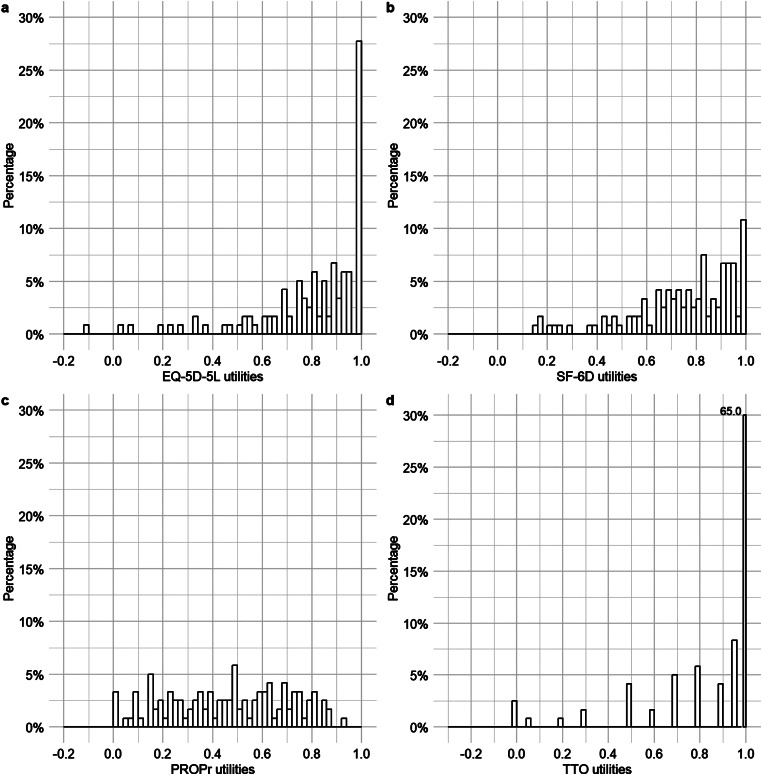
Distribution of EQ-5D-5L, PROPr, SF-6D and TTO utilities

In line with our hypotheses, the TTO produced the highest, while the PROPr resulted in the lowest average utilities. In the total sample, median (IQR) TTO, EQ-5D-5L, SF-6D and PROPr utilities were 1.00 (0.90-1.00), 0.87 (0.72-1.00), 0.81 (0.66–0.92) and 0.48 (0.26–0.68), respectively. The EQ-5D-5L and TTO utilities demonstrated a skewed distribution towards full health, while the PROPr and SF-6D exhibited rather symmetrical distributions (Fig. 1). The TTO showed a large (65.0%) and the EQ-5D-5L a small (27.5%) ceiling effect, but not the SF-6D (1.7%) and PROPr (0.0%) No floor effect was identified for any of the utility assessment methods (Table 2).

**Table 2:** Descriptive characteristics of utilities

Health utilities	Mean ± SD	Median (IQR)	Minimum	Maximum	Floor *N* (%)	Ceiling *N* (%)	psoriasis *N* = 47	atopic dermatitis *N* = 32	acne *N* = 23	other skin diseases *N* = 24
EQ-5D-5L	0.79 ± 0.25	0.87 (0.72-1.00)	-0.24	1.00	0 (0.00%)	33 (27.50%)	0.88 (0.68-1.00)	0.89 (0.66-1.00)	0.82 (0.74–0.90)	0.93 (0.72-1.00)
SF-6D	0.76 ± 0.21	0.81 (0.66–0.92)	0.15	1.00	0 (0.00%)	2 (1.70%)	0.79 (0.66–0.90)	0.83 (0.66–0.93)	0.74 (0.66–0.87)	0.80 (0.64–0.96)
PROPr	0.47 ± 0.24	0.48 (0.26–0.68)	0.01	0.94	0 (0.00%)	0 (0.00%)	0.48 (0.24–0.64)	0.57 (0.27–0.72)	0.35 (0.15–0.50)	0.46 (0.30–0.73)
TTO	0.89 ± 0.23	1.00 (0.90-1.00)	0.00	1.00	3 (2.50%)	78 (65.00%)	1.00 (0.80-1.00)	1.00 (0.90-1.00)	1.00 (0.95-1.00)	1.00 (0.73-1.00)

IQR = Interquartile range; PROPr = Patient-Reported Outcomes Measurement Information System-Preference; SF-6D = Short-form-6D; TTO = time trade-off

### Convergent validity and agreement

**Fig. 2:**
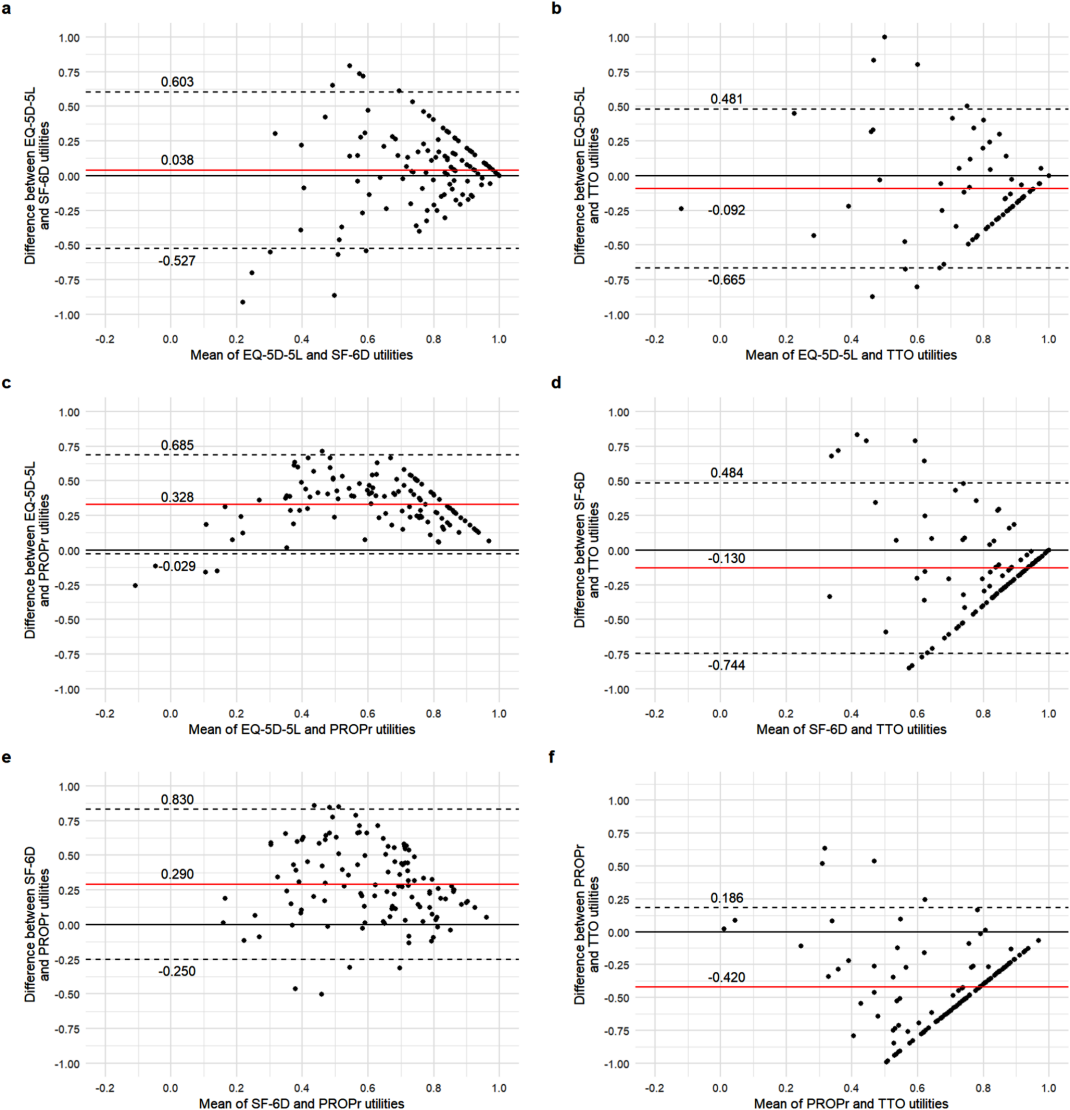
Bland-Altman plots of the utility values. The horizontal red line represents the mean of the differences between utility values, while the 95% limits of agreement, obtained as mean difference ± 1.96 *SD of mean difference, are indicated by dashed lines

Most hypotheses regarding the convergent validity and agreement of the utility values were met (Table 3). PROPr demonstrated poor agreement with the EQ-5D-5L (ICC = 0.381) and fair with SF-6D utilities (ICC = 0.445). TTO utilities showed poor agreement with indirectly assessed utilities (ICC = 0.058–0.242). In contrast to our expectations, the SF-6D showed excellent agreement with the EQ-5D-5L (ICC = 0.770). These results were also supported by the Bland-Altman plots, which indicated increasing differences between utilities towards the lower end of the scale (Fig. 2).

**Table 3:** Spearman’s correlations and intraclass correlations between health utilities

	EQ-5D-5L			SF-6D			PROPr		
	rho	ICC	95% CI	rho	ICC	95% CI	rho	ICC	95% CI
SF-6D	0.783	0.770	0.681–0.836	-	-	-	-	-	-
PROPr	0.771	0.381	-0.094–0.711	0.864	0.445	-0.081–0.773	-	-	-
TTO	0.180	0.242	0.072–0.399	0.143*	0.201	0.029–0.363	0.171*	0.058*	-0.052–0.185

CI = confidential interval; ICC = intraclass correlation coefficient; PROPr = Patient-Reported Outcomes Measurement Information System-Preference; rho = Spearman’s correlation coefficient; SF-6D = Short-form-6D; TTO = time trade-off

*All Spearman’s correlation and intraclass correlations coefficients were significant (*p* < 0.05), except in the cases marked with an asterisk

### Validity between known groups

The range of effect sizes was 0.187–0.432 for the EQ-5D-5L, 0.294–0.393 for the SF-6D, 0.249–0.326 for PROPr and 0.004–0.079 for the TTO (Table 4). All three indirect utility assessment methods showed large effect sizes for all known groups, while the TTO demonstrated moderate ES for physical health (Global03), and small ESs for general health (SF-36 first question) and quality of life (Global02). The TTO was not able to differentiate between respondents based on general health (Global01) and mental health (Global04) groups. In line with our hypotheses, the EQ-5D-5L was able to better discriminate between known groups of patients defined based on general health (SF-36 first question or Global01) and physical health than any other method. For quality of life groups, the SF-6D showed higher relative efficiency than the EQ-5D-5L (1.151), but not the PROPr or TTO. For mental health groups, both the PROPr and SF-6D outperformed the EQ-5D-5L with relative efficiencies of 1.513 and 1.571, but not the TTO.

**Table 4:** Known-groups validity of the health utilities, median (IQR)

	%	EQ-5D-5L	SF-6D	PROPr	TTO		
**General health (SF-36, first question)**							
Excellent and very good	27 (22.5%)	1.00 (0.88-1.00)	0.91 (0.80–0.99)	0.61 (0.46–0.79)	1.00 (1.00–1.00)		
Good	50 (41.7%)	0.89 (0.82-1.00)	0.84 (0.74–0.92)	0.55 (0.38–0.69)	1.00 (0.95-1.00)		
Poor	30 (25.0%)	0.73 (0.60–0.89)	0.72 (0.55–0.83)	0.34 (0.18–0.56)	1.00 (0.80-1.00)		
Very poor	13 (10.8%)	0.33 (0.13–0.58)	0.44 (0.19–0.53)	0.09 (0.02–0.23)	0.80 (0.40-1.00)		
p-value ^α^	-	**< 0.001**	**< 0.001**	**< 0.001**	**0.041**		
Kruskal-Wallis H	-	53.072	46.048	40.848	8.273		
Effect size	-	0.432	0.371	0.326	0.045		
Relative efficiency	-	-	**0.860**	**0.756**	**0.105**		
**General health (PROMIS Global01)**							
Excellent and very good	32 (26.7%)	1.00 (0.85-1.00)	0.91 (0.80–0.99)	0.61 (0.46–0.78)	1.00 (0.95-1.00)		
Good	48 (40.0%)	0.90 (0.79-1.00)	0.85 (0.73–0.93)	0.55 (0.35–0.70)	1.00 (0.91-1.00)		
Poor	29 (24.2%)	0.75 (0.62–0.84)	0.69 (055-0.76)	0.34 (0.19–0.46)	1.00 (0.85-1.00)		
Very poor	11 (9.2%)	0.45 (0.07–0.68)	0.47 (0.17–0.60)	0.15 (0.07–0.25)	0.90 (0.50-1.00)		
p-value ^α^	-	**< 0.001**	**< 0.001**	**< 0.001**	0.321		
Kruskal-Wallis H	-	44.389	44.136	34.402	3.496		
Effect size	-	0.357	0.355	0.271	0.004		
Relative efficiency	-	-	**0.994**	**0.759**	**0.012**		
**Quality of life (PROMIS Global02)**							
Excellent and very good	29 (24.2%)	1.00 (0.91-1.00)	0.94 (0.84–0.99)	0.70 (0.55–0.78)	1.00 (1.00–1.00)		
Good	60 (50.0%)	0.88 (0.75–0.94)	0.81 (0.69–0.91)	0.48 (0.27–0.64)	1.00 (0.91-1.00)		
Poor	22 (18.3%)	0.73 (0.60–0.88)	0.60 (0.47–0.75)	0.37 (0.18–0.48)	1.00 (0.80-1.00)		
Very poor	9 (7.5%)	0.33 (-0.02-0.74)	0.47 (0.17–0.80)	0.15 (0.05–0.37)	0.90 (0.15-1.00)		
p-value ^α^	-	**< 0.001**	**< 0.001**	**< 0.001**	**0.028**		
Kruskal-Wallis H	-	34.584	39.342	31.853	9.098		
Effect size	-	0.272	0.313	0.249	0.053		
Relative efficiency	-	-	**1.151**	**0.914**	**0.193**		
**Physical health (PROMIS Global03)**							
Excellent and very good	26 (21.7%)	1.00 (0.94-1.00)	0.92 (0.80–0.99)	0.65 (0.45–0.80)	1.00 (0.94-1.00)		
Good	47 (39.2%)	0.91 (0.84-1.00)	0.85 (0.77–0.94)	0.57 (0.44–0.71)	1.00 (0.95-1.00)		
Fair	31 (25.8%)	0.78 (0.63–0.84)	0.69 (0.55–0.81)	0.34 (0.16–0.55)	1.00 (0.95-1.00)		
Poor	16 (13.3%)	0.49 (0.11–0.67)	0.44 (0.23–0.65)	0.18 (0.09–0.27)	0.85 (0.35-1.00)		
p-value ^α^	-	**< 0.001**	**< 0.001**	**< 0.001**	**0.007**		
Kruskal-Wallis H	-	51.422	48.579	37.124	12.212		
Effect size	-	0.417	0.393	0.294	0.079		
Relative efficiency	-	-	**0.941**	**0.705**	**0.190**		
**Mental health (PROMIS Global04)**							
Excellent and very good	44 (36.7%)	0.94 (0.84-1.00)	0.91 (0.81–0.96)	0.62 (0.48–0.76)	1.00 (0.96-1.00)		
Good	40 (33.3%)	0.83 (0.63–0.94)	0.74 (0.57–0.90)	0.42 (0.25–0.64)	1.00 (0.80-1.00)		
Fair	26 (21.7%)	0.83 (0.65–0.94)	0.74 (0.65–0.87)	0.41 (0.19–0.55)	1.00 (0.80-1.00)		
Poor	10 (8.3%)	0.58 (0.31–0.73)	0.54 (0.21–0.58)	0.10 (0.05–0.24)	1.00 (0.68-1.00)		
p-value ^α^	-	**< 0.001**	**< 0.001**	**< 0.001**	0.314		
Kruskal-Wallis H	-	24.674	37.058	35.797	3.554		
Effect size	-	0.187	0.294	0.283	0.005		
Relative efficiency	-	-	**1.571**	**1.513**	**0.026**		

% = Numbers of patients; Promis-GH = Patient-Reported Outcomes Measurement Information System Global Health; PROPr = Patient-Reported Outcomes Measurement Information System-Preference; SF-6D = Short-form-6D; TTO = Time trade-off

^α^ The differences between known groups were tested by Kruskal Wallis tests, where a *p* < 0.05 was considered statistically significant. Relative efficiency compared to the EQ-5D-5L (US)

### Sensitivity analysis with the Hungarian EQ-5D-5L value set

The majority of the results were very similar when using the US and Hungarian value sets. Mean EQ-5D-5L utilities were somewhat higher with the Hungarian value set compared to the US one, both in the total sample and subgroups. The correlations, ICC values and effect sizes were slightly higher with the US value set, except for the mental health PROMIS Global04 item, where the effect size was higher with the Hungarian value set.

## Discussion

Our study compared the measurement properties of three indirect (EQ-5D-5L, PROPr and SF-6D) and a direct (TTO) utility assessment method in patients with chronic dermatological conditions. This is the first study in the literature to comparatively examine four different health utility assessment methods in chronic skin diseases. All indirect health utility assessment methods performed generally well in this population. The EQ-5D-5L showed the highest ceiling among the three indirect measures. All three methods were efficient in distinguishing between known groups of patients. In contrast, the TTO performed quite poorly, exhibiting an exceptionally high ceiling effect, very weak correlations and agreement with the other methods, and an inability to differentiate across most known groups.

The EQ-5D-5L exhibited higher effect sizes than PROPr and SF-6D across general health and physical health groups, while the SF-6D outperformed the EQ-5D-5L for quality of life and mental health, and the PROPr for mental health groups. The better performance of the PROPr and SF-6D for mental health may be explained by both descriptive as well as value set related characteristics of the instruments. The EQ-5D-5L’s descriptive system uses clinical terms such as ‘anxiety’ and ‘depression’ to evaluate mental health, alongside a severity scale. Conversely, the depression domain of PROMIS-29+2 includes items, such as ‘worthless’, ‘helpless’ or ‘hopeless’, while the SF-6D’s mental health item inquires about ‘feeling tense or downhearted and low’, both using a frequency scale that may enhance reporting of health problems. From the valuation side, it is notable that using the US value sets, the depression domain in PROPr and the mental health domain in SF-6D are ranked as the second most important out of the seven and six domains, respectively, in terms of the disutilities associated with the most severe levels. In comparison, in the EQ-5D-5L, the anxiety/depression domain is ranked as only the third most important out of the five.

With the exception of PROPr, all utility assessment methods tested in this study have been previously used or validated in various areas of dermatology [17, 18, 24, 26]. However, the EQ-5D-5L stands out as the most validated and widely used among these measures. The EQ-5D-5L has demonstrated good validity in several chronic dermatological conditions such as psoriasis, atopic dermatitis, hidradenitis suppurativa and pemphigus [32–34, 68]. Despite this, the content validity and responsiveness of the EQ-5D-5L may be limited in certain skin conditions, particularly psoriasis. To address this limitation, two additional ‘bolt-on’ items were developed for the EQ-5D-5L to improve its measurement performance in psoriasis, focusing on skin irritation and self-confidence [69–71]. This instrument also has a value set allowing the computation of utilities [70]. However, it was developed as a pilot value set before the EQ-VT protocol.

The TTO method used in the present study differs from indirect utility assessment methods in that the perspective of assessment was individual rather than societal, and respondents evaluated their own health rather than hypothetical health states as in the value set developments of the three generic preference-accompanied measures used in this study. The TTO method, which performed poorly in the present study, has previously been employed in a large number of chronic skin conditions, both to measure the current health of patients and to elicit utilities for hypothetical health state vignettes by members of the general public or patients [19, 72–79]. In our study, the mean TTO value was 0.89, which aligns with values reported for current own health in clinical populations, such as acne (0.96), atopic dermatitis (0.93), psoriasis (0.93) and melasma (0.92) [75, 77, 80, 81]. The high ceiling effect and its consequences are likely attributable to the overall good health status of the population, originating from a general population sample as well as the TTO approach used. In our study, only one health state (own current health) was valued in a single question, and no specific software or interviewer was used, which are known as limitations of the method [82]. Furthermore, the smallest tradable amount of time was 6 months, which may be too large considering the good health of the sample. While the usefulness of assessing dermatological patients’ own health by TTO is very limited based on our findings, TTO valuation of hypothetical health state vignettes may be useful in certain chronic skin conditions where typical generic health status measures lack relevant domains to sufficiently capture the health impacts of the condition and its treatment [83].

Our findings can be useful to inform decisions about the choice of instrument for cost-utility analysis. On the one hand, the EQ-5D-5L showed the best performance among the three indirect methods, it has the most national value sets available and it is also the shortest among the three questionnaires (5 items) [84]. However, SF-6D and PROPr performed somewhat better in differentiating between mental health aspects that are very often impacted in chronic skin conditions likely due to the adversely affected bodily appearance (e.g. low self-confidence, feeling of shame, anxiety, depression, body dysmorphic disorder) [85]. In general, PROPr is less recommended, given that it is an emerging new instrument that has no sufficiently established validity presently, and some previous studies also raised concerns about the face validity of unusually low PROPr utilities, which was the case also in our study (mean 0.47). Furthermore, the PROPr is the longest among these questionnaires, requiring at least 14 items to complete, increasing the cognitive burden for respondents [27, 59].

It is important to acknowledge some limitations of this study. Firstly, the sample size was relatively small, which limited the ability to conduct subgroup analyses for the measurement properties. However, it was sufficient to detect large effect sizes across all indirect measures when differentiating between known groups. Secondly, due to the online panel-based data collection, objective information on the patients’ disease severity and clinical status was unavailable, and the majority of respondents were in generally good overall health. We also lacked detailed information about the treatment status of our population. Therefore, the findings of this study may not be fully generalizable to specific patient groups with varying degrees of severity or treatment status. Thirdly, items from other HRQoL instruments were selected to define known groups, which were not based on clinical severity. Although our approach may only be considered second-best after using clinically-verified known groups, a similar approach has been used in several earlier psychometric testing studies of HRQoL and wellbeing measures [86, 87]. Fourthly, it is important to note that no skin-specific HRQoL instrument was used in our study, which could have been beneficial for testing the convergent validity. Fifthly, we used US value sets for all three indirect utility measures, as only the EQ-5D-5L has a national value set in Hungary [42]. As the aim of the study was a comparative assessment, we had to make a trade-off between using national or European or neighbouring countries’ value sets wherever possible and ensuring the consistency of preference weights used. However, as a sensitivity analysis, we repeated certain analyses using the Hungarian EQ-5D-5L value set to allow for a more direct comparison with TTO utilities derived from Hungarian patients. Across all measurement properties, including the agreement between EQ-5D-5L and TTO utilities, we observed only very minor differences with the Hungarian value set. Lastly, the cross-sectional nature of the study limited our ability to examine other measurement properties, such as test-retest reliability and responsiveness. These aspects should be the focus of future studies to provide a more comprehensive understanding of these utility assessment methods in the area of dermatology.

In conclusion, utilities measured by different instruments showed significant variability in a mixed sample of patients with chronic skin diseases, with the TTO utilities being the highest, followed by the EQ-5D-5L and SF-6D, while PROPr utilities being the lowest. All three indirect utility assessment methods (EQ-5D-5L, PROPr, SF-6D) showed sufficient validity in this population. The EQ-5D-5L appears to perform the best in terms of its ability to discriminate between known groups of patients. Future research should compare the measurement performance of these instruments across various patient populations and severity levels. Additionally, investigating the impact of instrument selection on cost-utility estimates would also be valuable.

## Electronic supplementary material

Below is the link to the electronic supplementary material.

Supplementary Material 1

## Data Availability

All data of this study are available from the corresponding author upon reasonable request.

## Code Availability

N/A.
