# Supplementary material for: Comparing EQ-5D-5L, PROPr, SF-6D and TTO utilities in patients with chronic skin diseases
Source: Eur J Health Econ. 2024 Sep 28;26(4):627–39. doi: 10.1007/s10198-024-01728-5 (PMC12126321; doi:10.1007/s10198-024-01728-5)
Supplement: Supplementary file 1 — Supplementary Material 1 [file 10198_2024_1728_MOESM1_ESM.docx]

**Electronic Supplementary Materials**

**Online Resource 1 Characteristics of the study population**

| Characteristics | Median (Q1-Q3) or n (%) |
| --- | --- |
| Total sample | 120 (100.0%) |
| Gender |  |
| Female | 73 (60.8%) |
| Male | 47 (39.2%) |
| Age (years) | 51 (34 - 65) |
| Employment status |  |
| Full-time employed | 39 (32.5%) |
| Part-time employed | 10 (8.3%) |
| Retired | 39 (32.5%) |
| Student | 7 (5.8%) |
| Disabled pensioner | 7 (5.8%) |
| Unemployed | 6 (5.0%) |
| Other | 12 (10.0%) |
| Highest level of education |  |
| Primary school or less | 36 (30.0%) |
| Secondary school | 43 (35.8%) |
| College / university degree | 41 (34.2%) |
| Place of residency |  |
| Other town | 60 (50.0%) |
| Village | 33 (27.5%) |
| Capital (Budapest) | 27 (22.5%) |
| Physician diagnosed chronic dermatological conditions ^a^ |  |
| psoriasis | 47 (39.2%) |
| atopic dermatitis | 32 (26.7%) |
| acne | 23 (19.2%) |
| other | 24 (20.0%) |
| EQ VAS (0-100) | 80.00 (69.25 - 89.00) |

EQ VAS = EuroQol Visual Analogue Scale

^a^ One respondent may have reported more than one condition.

**Online Resource 2 Sensitivity analysis of measurement properties between EQ-5D-5L utilities (computed with the Hungarian value set) and TTO utilities**

**Descriptive characteristics of utilities**

|  |  |  |  |  |  | chronic skin disease diagnosed by physician – Median (IQR) | | | |
| --- | --- | --- | --- | --- | --- | --- | --- | --- | --- |
| Health utilities | Mean (SD) | Median (IQR) | Minimum | Maximum | Ceiling effect,  N (%) *^α^* | psoriasis  N=47 | atopic dermatitis  N=32 | acne  N=23 | other skin diseases  N=24 |
| EQ-5D-5L (HUN) | 0.84 (0.22) | 0.92 (0.80-1.00) | -0.20 | 1.00 | 33 (27.50%) | 0.92 (0.80-1.00) | 0.92 (0.75-1.00) | 0.87 (0.80-0.92) | 0.96 (0.80-1.00) |
| Conventional TTO (10-year) | 0.89 (0.23) | 1.00 (0.90-1.00) | 0.00 | 1.00 | 78 (65.00%) | 1.00 (0.80-1.00) | 1.00 (0.90-1.00) | 1.00 (0.95-1.00) | 1.00 (0.73-1.00) |

**Spearman’s correlations and intraclass correlations between EQ-5D-5L (HUN) and TTO utilities**

The Spearman’s correlation coefficient between EQ-5D-5L (HUN) and TTO utilities was 0.193 (p<0.05). The intraclass correlations coefficient between EQ-5D-5L (HUN) and TTO utilities was 0.263 (95% confidence interval 0.090-0.420; p<0.05).

**Known-groups validity of the EQ-5D-5L (HUN) and TTO utilities [median (IQR)]**

|  | Numbers of patients (%) | EQ-5D-5L (HUN) | Conventional TTO (10-year) |
| --- | --- | --- | --- |
| **General health (SF-36, first question)** | | | |
| Excellent and very good | 27 (22.5%) | 1.00 (0.93-1.00) | 1.00 (1.00-1.00) |
| Good | 50 (41.7%) | 0.92 (0.88-1.00) | 1.00 (0.95-1.00) |
| Poor | 30 (25.0%) | 0.82 (0.73-0.92) | 1.00 (0.80-1.00) |
| Very poor | 13 (10.8%) | 0.40 (0.18-0.72) | 0.80 (0.40-1.00) |
| p-value ^α^ | - | <0.001 | 0.041 |
| Kruskal-Wallis H | - | 51.176 | 8.273 |
| Effect size | - | 0.415 | 0.045 |
| Relative efficiency | - | **-** | 0.110 |
| **General health (PROMIS Global01)** | | | |
| Excellent and very good | 32 (26.7%) | 1.00 (0.93-1.00) | 1.00 (0.95-1.00) |
| Good | 48 (40.0%) | 0.92 (0.88-1.00) | 1.00 (0.91-1.00) |
| Poor | 29 (24.2%) | 0.82 (0.73-0.92) | 1.00 (0.85-1.00) |
| Very poor | 11 (9.2%) | 0.40 (0.18-0.72) | 0.90 (0.50-1.00) |
| p-value ^α^ | - | <0.001 | 0.321 |
| Kruskal-Wallis H | - | 42.713 | 3.496 |
| Effect size | - | 0.342 | 0.004 |
| Relative efficiency | - | **-** | 0.012 |
| **Quality of life (PROMIS Global02)** | | | |
| Excellent and very good | 29 (24.2%) | 1.00 (0.93-1.00) | 1.00 (1.00-1.00) |
| Good | 60 (50.0%) | 0.92 (0.88-1.00) | 1.00 (0.91-1.00) |
| Poor | 22 (18.3%) | 0.82 (0.73-0.92) | 1.00 (0.80-1.00) |
| Very poor | 9 (7.5%) | 0.40 (0.18-0.72) | 0.90 (0.15-1.00) |
| p-value ^α^ | - | <0.001 | 0.028 |
| Kruskal-Wallis H | - | 32.378 | 9.098 |
| Effect size | - | 0.253 | 0.053 |
| Relative efficiency | - | **-** | 0.208 |
| **Physical health (PROMIS Global03)** | | | |
| Excellent and very good | 26 (21.7%) | 1.00 (0.96-1.00) | 1.00 (0.94-1.00) |
| Good | 47 (39.2%) | 0.96 (0.92-1.00) | 1.00 (0.95-1.00) |
| Fair | 31 (25.8%) | 0.85 (0.74-0.89) | 1.00 (0.95-1.00) |
| Poor | 16 (13.3%) | 0.60 (0.15-0.81) | 0.85 (0.35-1.00) |
| p-value ^α^ | - | <0.001 | 0.007 |
| Kruskal-Wallis H | - | 51.047 | 12.212 |
| Effect size | - | 0.414 | 0.079 |
| Relative efficiency | - | **-** | 0.192 |
| **Mental health (PROMIS Global04)** | | | |
| Excellent and very good | 44 (36.7%) | 0.97 (0.92-1.00) | 1.00 (0.96-1.00) |
| Good | 40 (33.3%) | 0.90 (0.77-0.96) | 1.00 (0.80-1.00) |
| Fair | 26 (21.7%) | 0.89 (0.80-0.96) | 1.00 (0.80-1.00) |
| Poor | 10 (8.3%) | 0.70 (0.43-0.83) | 1.00 (0.68-1.00) |
| p-value ^α^ | - | <0.001 | 0.314 |
| Kruskal-Wallis H | - | 26.274 | 3.554 |
| Effect size | - | 0.201 | 0.005 |
| Relative efficiency | - | - | 0.024 |

^α^ The differences between known groups were tested by Kruskal Wallis tests, where a p < 0.05 was considered statistically significant. Relative efficiency compared to the EQ-5D-5L (HUN)

**Bland-Altman plot of the EQ-5D-5L (HUN) and TTO utilities**


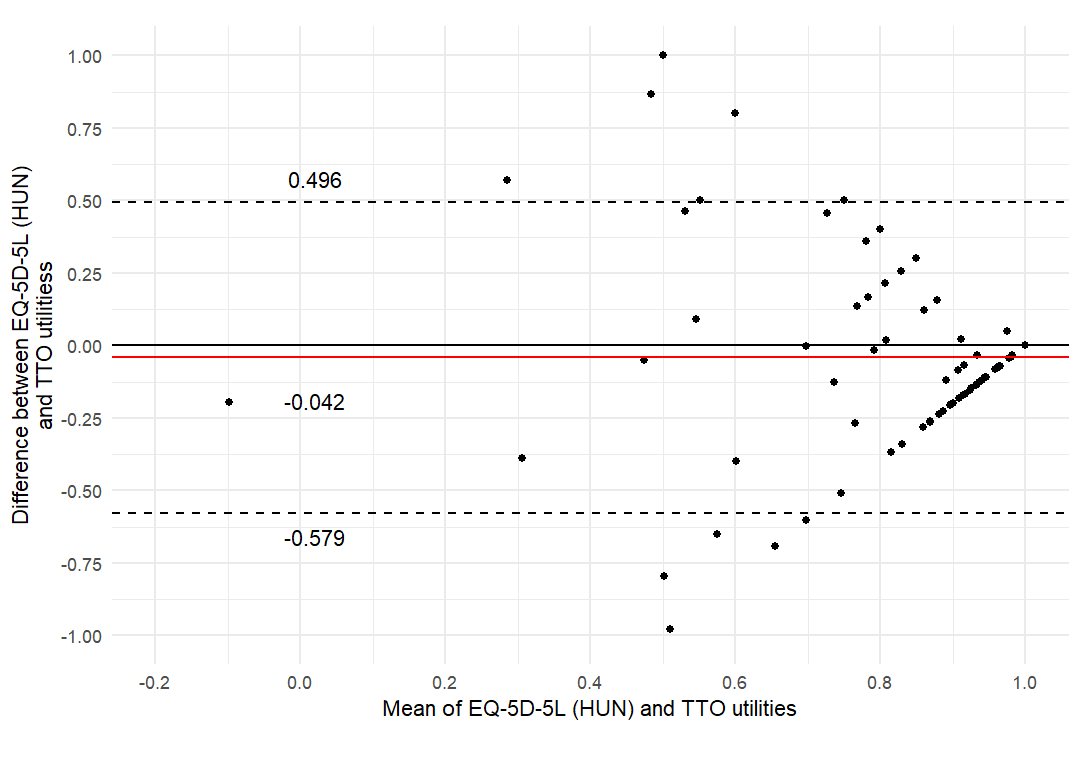


The horizontal red line represents the mean of the differences between utility values, while the 95% limits of agreement, obtained as mean difference ± 1.96 *SD of mean difference, are indicated by dashed lines.

TTO = Time trade-off
